# Dietary Polyphenols and Their Role in Gut Health

**DOI:** 10.3390/nu15122650

**Published:** 2023-06-06

**Authors:** Nadia Calabriso, Marika Massaro, Egeria Scoditti, Maria Annunziata Carluccio

**Affiliations:** National Research Council (CNR), Institute of Clinical Physiology (IFC), Campus Ecotekne, Via Monteroni, 73100 Lecce, Italy; marika.massaro@ifc.cnr.it (M.M.); egeria.scoditti@ifc.cnr.it (E.S.); maria.carluccio@ifc.cnr.it (M.A.C.)

Polyphenols are secondary plant metabolites derived from the shikimate/phenylpropanoid pathway, protecting plants from physical, chemical and biological stress. They are the most common phytochemical bioactive components in our diet, being present in a variety of foods such as fruits, vegetables, grains and legumes; beverages such as tea, coffee, chocolate and wine; and in extra virgin olive oils. Polyphenols exhibit multiple health-promoting activities due to their antioxidant, anti-inflammatory and immunomodulatory properties which contribute to prevent the onset and progression of various chronic inflammatory diseases.

The intestine is a prime target for polyphenols. Although they have low bioavailability in the bloodstream, polyphenols can be retained in the gut for a longer time due to their complex structure and food matrix composition, reaching local high concentrations, thus promoting beneficial gut effects. Growing evidence suggests that polyphenols exhibit prebiotic properties and antimicrobial activities against pathogenic gut microflora, in addition to modulating gut metabolism and immunity and displaying anti-inflammatory effects.

The Special Issue “Dietary Polyphenols and Their Role in Gut Health” has collected eight papers, including three scientific literature reviews and five original research articles, concerning the impact of polyphenols on intestinal health.

In particular, a review by Shabbir et al. [1] has provided an overview of the biological role of polyphenols in counteracting metabolic disorders such as obesity, diabetes, cancer and cardio-metabolic disorders, improving gut health. An in-depth description of the biotransformation of curcumin, quercetin and catechins by gut microbiota and the beneficial effects of intestinal bioactive metabolites on the composition of the intestinal microbiota have been provided. Furthermore, given the low bioavailability of polyphenols, a section of the review was dedicated to the analysis of various approaches developed to improve the solubility and transport of polyphenols through the intestine.

Another review by Stompor-Gorący et al. [2] also addressed the low bioavailability of polyphenols with a particular reference to chrysin, a dietary dihydroxyflavone endowed with various biological activities, including the prevention of oxidative stress, inflammation, neurodegeneration and carcinogenesis. Due to poor water solubility, numerous attempts to functionalize chrysin have been undertaken to increase absorption, bioavailability and improve in vivo delivery. The review summarized the most recent research on chrysin, including its sources, metabolism, pro-health properties and effects of its functionalization on biological activity and pharmacological efficacy (evaluated both in vitro and in vivo).

Our research group has provided an updated overview of the effects of polyphenols as possible dietary strategies to counteract the toxic effects of gluten, with the potential to improve the quality of life of patients with gluten-related disorders [3]. The metabolic fate of food polyphenols, both as free forms and bound to macromolecules, has been described, with particular reference to the gastrointestinal compartment. In addition, potential targets of polyphenols were outlined, including gluten peptide bioavailability, intestinal epithelial barrier dysfunction, intestinal immune response, oxidative stress, inflammation and dysbiosis.

Two of the published as original research articles focused on the health effects of hydroxycinnamic acids [4,5]. A study by Zielińska et al. [4] shed some light about the chemistry of caffeic acid and the molecular mechanisms associated with its anti-inflammatory effects at the intestinal level, using IL-1β-stimulated colonic myofibroblasts as a relevant human model of intestinal inflammation.

Another polyphenol that belongs to the group of hydroxycinnamic acids is ferulic acid, present in numerous vegetable foods, including cereals. The dietary consumption of ferulic acid leads to the formation of various phase I and II metabolites, which represent the prevalent form of ferulates in the circulation and the intestine. Serreli et al. [5] reported that the major metabolites of ferulic acid, including isoferulic acid, dihydroferulic acid, ferulic acid glucuronide, dihydroferulic acid glucuronide and isoferulic acid sulfate, retained the efficacy of their free dietary form in counteracting the inflammatory response in intestinal epithelial cells, thus hampering or limiting the progression of intestinal inflammation and related diseases.

Following the ingestion of foods and fruits rich in ellagitannins, such as pomegranates, grapes, nuts, and berries, the gut microbiota produces secondary metabolites, such as urolithins, including urolithin A and B. These urolithin metabolites are more bioavailable than the parent polyphenol compounds. They have been detected in various target tissues and therefore have been suggested as responsible for the biological activities related to the intake of foods containing ellagitannins. In particular, urolithin A has recently been approved as a functional food ingredient. An in vivo study by Al Khalaf et al. [6] examined the impact of the administration of urolithin A and urolithin B on a metabolically unchallenged state in rats fed on a normal diet. The authors showed that both urolithins A and B did not affect weight gain in normal diet-fed rats. However, these metabolites enhanced liver and kidney functions. Although urolithins A and B exhibited differential impacts on gut microbiota composition, both urolithins induced the growth of Akkermensia and increased the abundance of Bdellovibrionales, two important microbes which have positive impacts on different metabolic diseases and on the control of intestinal pathogens, respectively.

Finally, two research articles recognized the by-products of olive oil and red wine production as valuable polyphenol-rich sources that could be used, in the circular economy, as nutraceuticals to maintain intestinal health by preventing chronic inflammatory bowel disease [7,8]. Indeed, waste represents a cost for companies, particularly for agri-food companies, which can become a resource as a secondary material, rich in bioactive compounds such as polyphenols.

The research paper by Curci et al. [7] addresses the biological activity of polyphenols from olive-mill wastewater with a focus on irritable bowel syndrome (IBS). Three products of olive-oil wastewater, named MOMAST^®^ (Plus30, PW25 and HY100), have been examined by analyzing their effects on some targets linked to IBS such as antioxidant action and spontaneous and induced intestinal contractility of the ileum and colon. In particular, the findings showed that Plus30 exhibited the most interesting effects on IBS targets due to its high concentration of polyphenols and oleuropein, highlighting the ability of Plus30 to modulate spontaneous and induced contractility, to exert a good antioxidant effect, and significantly act on various microorganisms. These effects were synergistic in the presence of antibiotics, confirming that Plus30 could be a great candidate as a food supplement in patients with IBS.

Furthermore, the article by Calabriso et al. [8] proposes grape pomace as a natural source of polyphenols with multiple health-promoting properties that could contribute to mitigating gut chronic inflammatory diseases and improve vascular endothelial function. The authors showed that grape pomace polyphenolic extract supplementation prevented, in a concentration-dependent manner, the stimulated intestinal expression and release of multiple pro-inflammatory mediators including IL-6, IL-1β, TNF-α and matrix metalloproteinases MMP-9 and MMP-2 by exhibiting ROS scavenging ability and inhibiting the activation of redox-sensitive transcription factors. Moreover, the crosstalk between intestinal and endothelial cells was investigated by analyzing the transepithelial effect of grape pomace polyphenolic extract on endothelial dysfunction. The results showed that transepithelial grape pomace polyphenolic metabolites suppressed the endothelial expression of adhesion molecules (VCAM-1 and ICAM-1) and the subsequent adhesion of leukocytes to the endothelial cells under pro-inflammatory conditions. These results suggest that grape pomace polyphenols may blunt the overwhelming inflammatory response both at an intestinal and vascular level with potential health-promoting properties in inflammatory bowel disease, which is characterized by the chronic inflammation of the gastrointestinal tract, combined with systemic vascular manifestations.

Collectively, the papers included in this research topic confirm the health benefits of polyphenols and provide novel insights into the impact of dietary polyphenols or their metabolites on gut health, contributing to microbiota/host equilibrium, well-being promotion and the prevention of chronic degenerative diseases.
